# Does changing to brighter road lighting improve road safety? Multilevel longitudinal analysis of road traffic collision frequency during the relighting of a UK city

**DOI:** 10.1136/jech-2019-212208

**Published:** 2020-05-01

**Authors:** Paul Marchant, James David Hale, Jon Paul Sadler

**Affiliations:** 1 Leeds Beckett University, Headingley Campus, Leeds, UK; 2 LICAMM, University of Leeds, Leeds, West Yorkshire, UK; 3 Institute of Ecology and Evolution, University of Bern, Bern, Switzerland; 4 The School of Geography, Earth and Environmental Sciences, The University of Birmingham, Birmingham, UK

**Keywords:** traffic, injuries, multilevel modelling

## Abstract

**Background:**

A step change in the night environment is taking place, with the large-scale installation of bright, broad-spectrum road lighting such as white light-emitting diodes (LEDs). One justification for this is a reduction in road traffic collisions (RTCs). This study aimed to estimate the effect of new lighting on personal injury RTCs within a large UK city.

**Methods:**

We analysed a 9-year time series of weekly RTC personal injury counts in 132 areas of the city using multilevel modelling. The RTC rate over a full 24-hour period was the primary outcome; darkness and daylight RTC rates were secondary. The background change in RTC rate was separated from the change associated with the number of newly installed bright lamps by including a polynomial underlying time trend for the logarithm of the mean number of collisions per week for each area. The study was based on a rigorous, predesigned and archived protocol.

**Results:**

Within-area coefficients for the broad lighting effect were positive; as the number of bright lamps in an area increased, so did the RTC rate. The estimate for the increase in the within-area 24-hour RTC rate is 11% (95% CI 2% to 20%). The estimate of darkness-only RTCs is 16% (95% CI 2% to 32%). If the effect of lighting on darkness RTC rate is adjusted by that for daylight, one obtains 4% (95% CI −12% to +23%).

**Conclusion:**

No evidence was found for bright lamps leading to an improvement in road safety in any of the analyses. For this city, introducing brighter road lighting may have compromised safety rather than reducing harm.

## Introduction

Worldwide, large-scale shifts in street lighting technology are under way, characterised by the replacement of mercury and sodium-based lamps with white light-emitting diodes (LEDs). Motivations include cost and carbon emission savings,^1 2^ supported by new sustainability regulations.^3^ Reductions in crime and road collisions are also used to support business cases for large-scale lamp replacement.^4–6^

**Figure 1:**
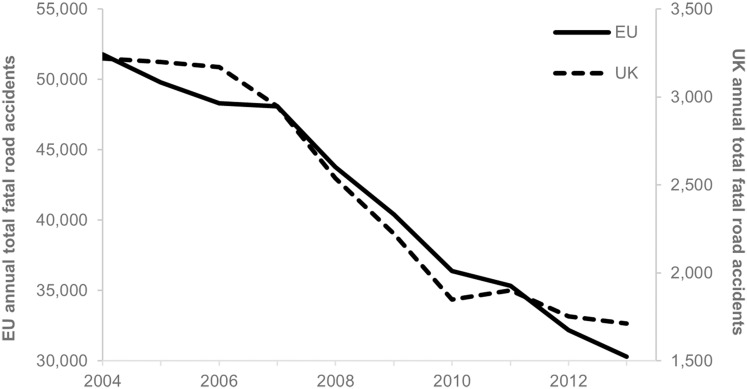
Annual road fatality totals for 28 countries of the EU (unbroken line) and for the UK (dashed line). EU, European Union.

Injuries from road traffic collisions (RTCs) are a major global public health concern. RTCs are the leading global cause of death by injury and are predicted to be in the top five causes of all deaths by 2030.^7^ Within the European Union (EU) and UK, there has been a long-term decline in deaths due to road collisions (figure 1).^8 9^ Key risk factors include excessive speed, drink driving and failure to use either seatbelts, child restraints or motorcycle helmets.^10^ Given this long-term decline in fatal collisions within the EU and the multiple causes of RTCs, identifying the additional impact from changes to street lighting requires a careful and robust statistical approach.

The systematic review of the impact of lighting on road safety by Beyer *et al* ^11^ identified 145 potentially relevant studies, of which just 17 met their screening criteria for inclusion. In addition, the review points out that ‘the methodological quality of the included studies was generally poor’. The conclusion of the review that road lighting may improve road safety has been criticised (see the Feedback section at end of the review and also at https://understandinguncertainty.org/node/231). The degree to which changes to road lighting have a practically relevant impact on collision risk remains an open question which this study aims to help resolve. Typical deficiencies in road lighting studies include a lack of evidence that they are robust against publication bias and regression towards the mean (RTM) (as in the above-mentioned critiques), or adequately account for underlying temporal trends in RTC risk. It is not clear that protocols for study design and analysis were written before studies commenced, as is typical in more regulated fields such as healthcare research (cf UN and Marchant^12 13^). A recent study of thousands of road collisions in England and Wales from 62 local authorities gave a null overall result from changing road lighting.^14^

The purpose of this paper is to present an enhanced approach to estimating the road safety impacts of large-scale street lighting replacement. For a case study city, we modelled the weekly number of personal injury RTCs with the increasing number of bright broad-spectrum lamps, as a function of time. This study monitors the change in the RTC rate in an area as the ‘dose’ of new lighting is increased at various time points while comparing with other areas where lighting is changed at different times and amounts. The underlying downward trend in collisions is fitted by a polynomial in time. This was achieved through a multilevel approach, which is appropriate for the structure of the data, as the RTCs and lamp changes, implemented in a ‘stepped wedge’ fashion, are nested within 132 neighbourhoods. The stepped wedged introduction of new lamps constitutes a sporadically interrupted time series. To maximise transparency and help guard against reporting bias, the protocol for the study (online supplementary file 1) was sent to independent custodians on 24 February 2015, and the analysis dataset is available to download from a public, open access repository.

10.1136/jech-2019-212208.supp1 Supplementary data

## Materials and methods

**Figure 2:**
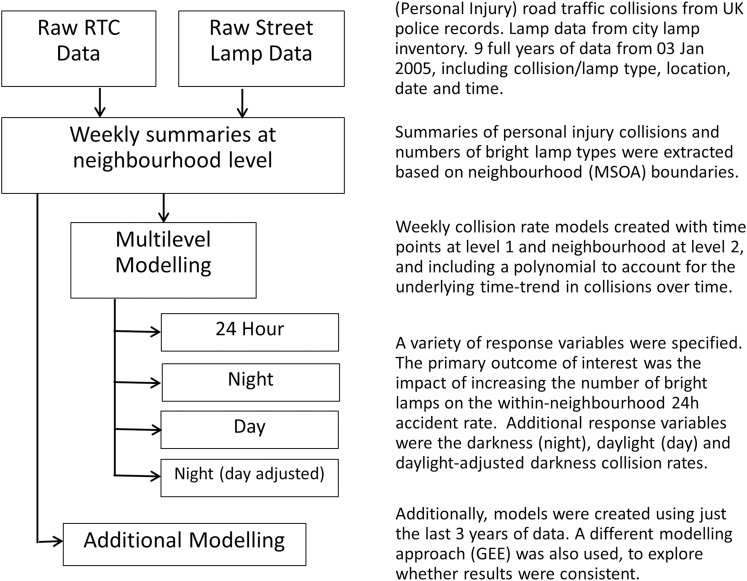
Key steps in in the analysis. MSOA, Middle Layer Super Output Area; RTC, road traffic collision; GEE, generalised estimating equation.

An overview of the key steps in our analysis is provided in figure 2.

### Lamp data

A full street lamp inventory for the city was made available for our analysis and screened for errors of three types: (1) *ghost* lamps, (2) lamps missing spatial data and (3) duplicate records (see the Methods section in online supplementary file 2 for details). Between Monday, 3 January 2005, and Sunday, 29 December 2013, only 61 *dull lamps* were installed as replacement lamps; 36 123 *bright white lamps* were installed over this period (see the Methods section in online supplementary file 2 for details and definitions). On 3 January 2005, 44 094 bright lamps were already in place. By 29 December 2013, the total for bright lamps had increased to 80 217, or 86.3% of the total street lamps. The majority of these bright lamps were high pressure sodium (HPS) (59 479) and LED (18 214). Weekly neighbourhood (MSOA) summaries of lamps (total dull and total bright) were generated between week 1 (starting Monday, 3 January 2005) and week 469 (starting Monday, 23 December 2013) (inclusive). This was undertaken in ArcGIS V.10.2 (ESRI, Redlands, California, USA), using the 2011 Middle Layer Super Output Area (MSOA) boundaries for the city. MSOAs are geographical units used in the UK to collect neighbourhood statistics (see the Methods section in online supplementary file 2 for details). The total number of RTCs within each MSOA was also summarised in a similar way.

10.1136/jech-2019-212208.supp2 Supplementary data

### Collision data

STATS19 data (personal injury collisions reported to the police) were sourced from the UK Department for Transport (http://data.gov.uk/dataset/road-accidents-safety-data). The STATS19 data contains a ‘lighting code’ for the reported state of natural lighting at the time of the collision (see online supplementary file 2); we assigned collisions a binary code of either *daylight* or *darkness*. Daylight is defined as starting 30 min before sunrise and ending 30 min after sunset; otherwise, it is darkness. A total of 20 282 daylight collisions and 8085 darkness collisions were recorded over the 9-year study period.

### Analysis dataset

The data in the analysis set consisted of the number of RTCs occurring each week (chosen in order to balance the varying traffic flows within a week) in each of the 132 MSOAs, together with the number of bright lamps operating in each MSOA. The time series ran from the week commencing Monday, 3 January 2005, to that ending Sunday, 29 December 2013 (the MSOAs form level 2 and the 469 weeks form level 1 of the multilevel analysis). There were no missing data.

### Descriptive statistics for the lamp data

**Figure 3:**
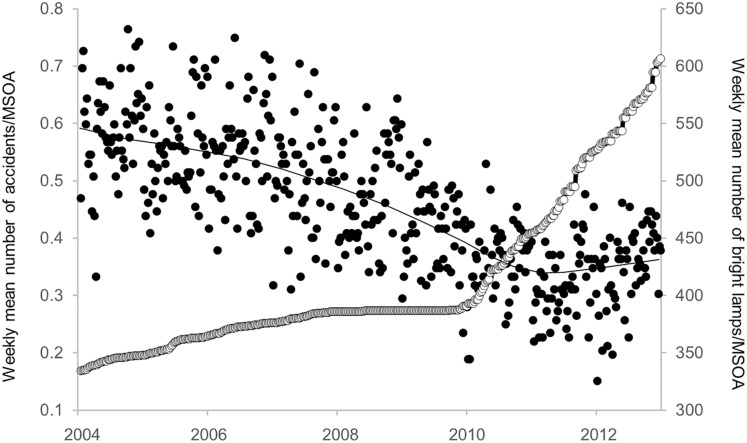
Scatter plot of the weekly mean number of RTCs/MSOA over the time period (black points) versus the weekly mean number of bright lamps/MSOA (white points). The solid line is an Epanechnikov (40%) smoother for the RTC data. MSOA, Middle Layer Super Output Area; RTC, road traffic collision.

The time series of the mean count of bright lamps, averaged over MSOAs, exhibits an increase over the period of study, with a sharp increase from 2010 (figure 3). This broad pattern is also evident at the level of individual MSOAs (see figure in online supplementary file 3). The increase in the numbers of bright lamps within the MSOAs over the analysis time period had minimum=9, maximum=680, mean=273.66, SD=149.77.

10.1136/jech-2019-212208.supp3 Supplementary data

### Descriptive statistics for the RTC rates

The mean weekly RTC rate, over the whole period, was calculated for each MSOA (ie, 132 mean rates). Statistics for these mean RTC rates are given in table 1. The RTC time series exhibits a general decline over the period of study (figure 3). The decline appears to cease after 2010. Further statistics on RTC counts are available in online supplementary file 2.

**Table 1:** Statistics for mean weekly RTC rate at the MSOA level for the whole analysis time period

	Minimum	Maximum	Mean	SD
Mean darkness MSOA RTC weekly rates	0.0043	0.5778	0.1306	0.0971
Mean daylight MSOA RTC weekly rates	0.0597	1.4755	0.3276	0.2178
Mean 24-hour MSOA RTC weekly rates	0.0640	2.0533	0.4582	0.3108

MSOA, Middle Layer Super Output Area; RTC, road traffic collision.

### Multilevel modelling

The protocol for the study (online supplementary file 1) was sent to independent custodians on 24 February 2015. The primary outcome used is the number of RTCs occurring at any time of the day during a particular week, in a given MSOA. This measure was chosen because we were testing what happens to the RTC rate when bright lamps were introduced. Analyses were also carried out for darkness and daylight only RTC rates, separately, also allowing the ratio of darkness to daylight RTCs to be obtained.

We analysed weekly counts of RTCs in each MSOA as a multilevel model: time points at level 1 and MSOA at level 2, for the logarithm of the mean number of collisions per week in the MSOAs, using a polynomial for the underlying time trend. We included a measure of the amount of new bright lighting introduced, which in the primary analysis was simply the number of new bright lamps operating each week in a MSOA. Indicator variables were included to reduce background effects on the RTC rate from seasonality and public holidays (see online supplementary file 2 for details).

The progress of the relighting was denoted as the difference in the number of bright lamps within each area from its mean over the series. The models also included a second lighting term for the difference of a MSOA’s mean number of bright lamps from the (grand) mean number across all MSOAs. The two terms for the build-up of bright lighting were thus ‘centred’. The aim of the modelling was to separate the underlying temporal change in RTC rate (generally decreasing) from the change associated with the brightening of street lighting.

The final form of the model used was

log(µ_ij_)=β_0_+β_1_t+β_2_t^2^+…+β_Mk_Month_k_+β_Hl_PubHol_l_+β_W_(L_ij_–<L_ij_>_j_)+β_B_(<L_ij_>_j_–<<L_ij_>>),

where <>_j_ denotes the mean with respect to week i in area j; <<>> denotes the mean of the area means; and t=the time that the midweek is from the winter solstice (21 December 2004) prior to the start of the series.

The β_0_ term, the intercept coefficient, was modelled as a random term because different areas will be differentially busy; β_1_t+ β_2_t^2^+… represents the underlying secular time trend, a polynomial with a degree to be determined. Polynomial coefficients, for example, β_1_, might also be expected to be random because of different temporal changes between different MSOAs. The β_W_ term represents the effect of the deviation of the number of bright lamps, L_ij_, from its mean <>, over the time series duration, in the area, giving the within-area effect of lighting change. This coefficient enables the effect of changing lighting to be seen. The β_B_ term is the between-area term, the effect of the deviation of the mean number of bright lamps in an area, over the series, from the mean of the MSOA means. The β_Mk_ term represents the effect of the k=1 to 11 month indicator variables (reference =January) and β_Hl_ that of the seven public holiday weeks per year l=1 to 7 (reference=weeks which are not public holiday weeks), the latter being the two ‘deviations from protocol’ made during the analysis (see the Protocol section in online supplementary file 1 and the Methods section in online supplementary file 2).

The models of the two secondary outcomes of separate darkness-only RTCs and daylight-only RTCs incorporated an offset; the logarithm of time exposure, that is, logarithm of the fraction of the 24-hour period that darkness or daylight applied.

The predictor variables, that is, time and number of lights, were scaled, in order to ensure that all coefficient values were of a convenient size (neither too big nor too small) in the output produced. The time variable, the number of weeks since the winter solstice of 2004, was scaled to be in decades and the number of bright lamps was put in units of 100.

The principal statistical modelling used a Poisson structure. Estimation was done using Markov Chain Monte Carlo (MCMC)^15^ within the package MLwiN 2.34.^16^ MCMC was used to improve estimates and also to obtain the information criterion in order to help select an appropriate model. The Poisson distribution was anticipated to be satisfactory, with model fit validated by examining the Pearson residuals. As planned, overdispersion was investigated, as was using the alternative, negative binomial distribution (see the Model validation section of the Results in online supplementary file 4).

10.1136/jech-2019-212208.supp4 Supplementary data

In order to mirror the approach adopted by others (eg, Perkins *et al*^14^) in which the darkness RTC rate is adjusted by the daylight rate, we also did this (see the Methods section in online supplementary file 2 and the Results section in online supplementary file 4). Taking this approach is said to compensate for changes in RTC rates due to changes in other features of the roads involved. We obtained an estimate of the lighting effect in darkness, adjusted for daylight RTC rate, by differencing the fitted darkness and daylight models. This was also investigated using other models.

The ratio of the citywide time-exposure compensated darkness to daylight raw rates was calculated in SPSS V.23.0^17^ and plotted against time (see figure in online supplementary file 5).

10.1136/jech-2019-212208.supp5 Supplementary data

In response to a reviewer comment, we explored adding to the primary analysis model, (1) an interaction between lighting and time, and (2) using a dummy variable to denote the intense relighting period from 2011 (both are deviations from the study protocol). Adding an interaction term yielded an estimate of the coefficient, which is small in magnitude and dwarfed by its standard error (SE), indicating that such a term is not needed in the final model (see the Results section in online supplementary file 4 for details). It is also possible that the effect of new lamps was different in the intense relighting period; therefore, as a check we included a dummy variable to denote the period of intense relighting. The change in estimate of the coefficient value was considerably smaller than the SE (see the Results section in online supplementary file 4 for details), again indicating that such a term is not needed in the model.

## Results

### The primary analysis (using the number of lamps)

The best models for 24 hours and daylight log RTC rate had the intercept, linear and quadratic temporal coefficients random and the cubic and quartic ones fixed. For the darkness model, all were fixed apart from the intercept. Lighting coefficients were positive for the three outcome measures: 24 hours, darkness and daylight. The confidence limits used are 95%. No effect was found for lighting in darkness when adjusted for the daylight RTC rate. The estimates relating to the key within-area effect of the number of bright lamps are given in table 2 with their SEs.

**Table 2:** Within-area effects of adding bright lamps for 24 hours, darkness and daylight when modelled using the number of bright lamps

	Within MSOA model coefficient for 100 bright lamps added	Within MSOA model estimate SE	Median RTC increase for 274 lamps replaced (%)	Lower confidence limit of the increase (%)	Upper confidence limit of the increase (%)
24 hours	0.0366	0.0159	11	2	20
Darkness	0.0549	0.0242	16	2	32
Daylight	0.0395	0.0193	11	0	24

The percentage increases are the effect on the RTC rate occurring for a 274 increase (the mean) in the number of bright lamps.

MSOA, Middle Layer Super Output Area; RTC, road traffic collision.

Within-area coefficients were positive, indicating that as the number of bright lamps increases within an area, so does its RTC rate. Exponentiating to obtain µ, rather than its log, to give the mean rate of collisions, gives a factor exp(β_w_(L_ij_–<L_ij_>_j_)). This leads to exp(β_w_(L_Ij_–L_1j_)) for the effect, µ_Ij_/µ_1j_, of bright lamps on the mean RTC rate at the end i=I=469 to that at the beginning i=1 . The results suggest that for areas with a typical increase in the number of bright lamps, the RTC rate is likely to be more than 10% over the expectation for no lighting change (see table 2).

Between-area coefficient estimates of β_B_ were all positive, 24 hours 0.0934 (0.0188), darkness 0.1088 (0.0224) and daylight 0.0954 (0.0185). These all indicate that more collisions occur in areas with a higher average number of bright lamps installed.

Analyses by other models (limiting the analysis to data from 2010 to 2013, and using a GEE approach) gave results very close to the above-mentioned analyses (see the Results section in online supplementary file 4).

### Adjusting for the rate of daylight RTCs

The lamp effect for log (µ_dark_/µ_daylight_)=0.0549–0.0395=0.0154. The associated SE is given by (0.0242^2^+0.0193^2^)^0.5^=0.0310 on the assumption of statistical independence. Thus, the SE of this log ratio of means is larger than its point estimate and therefore indicates no statistically significant difference from zero. The point estimate of change due to brightening in an area receiving the average number of brighter lamps is 4% in a 95% CI (−12% to +23%).

Other models, for example, binomial, were also run to estimate the RTC rate in darkness when adjusted by the daylight rate. These gave very similar results (section 6 of Supplementary information results in online supplementary file 4).

## Discussion

### Lighting replacement and the 24-hour, darkness and daylight RTC rates

The results show that the RTC rates (24 hours, darkness and daylight) increase in MSOAs receiving more new lamps. However, when the darkness rate is adjusted by the daylight rate (an often-used measure), there is no evidence of any lighting effect. This raises the question of whether the increased raw RTC rates are directly *caused* by new lighting (eg, through changes in driver behaviour), or due to extraneous factors, such as simultaneous changes to road layout or traffic flows. Future road-lighting research that concurrently measures traffic speeds and flows could therefore be useful.

### Methodical considerations/comparisons

Our study mainly focused on addressing the problem of underlying temporal trends in RTC risk, but we accept that there remain questions related to publication bias and RTM in this field. Our use of a protocol (shared with three independent academics) and the publication of our dataset potentially helped to guard against reporting or publication bias; there was an expectation by others that we would publish all results and that the analyses could be checked. However, greater community pressure might be exerted by placing future protocols on a public register. Regarding RTM, we consider this risk to be low due to the large fraction of street lamps replaced during the analysis timeframe. We have no evidence of any targeting of replacement lamps at accident hotspots, and such locations by definition would not be common. RTM is a much greater risk for small, poorly replicated, before–after studies, where high RTC locations may be selected, and where natural variation in RTC rate may be misinterpreted. If RTC spikes were the reason for lamp changes in our study, the positive correlation found between greater relighting and higher RTC rate is in the opposite direction to that resulting from regression to the mean, so the rise in the RTC rate with increasing number of new lamps cannot be an artefact of RTM.

Our work is similar to the ‘LANTERNS’ project of Perkins *et al*,^14^ the most rigorous and extensive (in space and time) study we are aware of. An aim stated in their protocol was to quantify the impact of street lighting reduction schemes in the UK on the incidence of road traffic injuries, later, in a variation, broadened to consider changes to white lights. When discussing our methodology, we have therefore drawn comparisons with their work, highlighting key differences with our approach in the following paragraphs. It is noteworthy that despite different approaches, our daylight adjusted result for the city of Birmingham (+4%, CI −12% to +23%) is similar to, but with a smaller CI than the LANTERNS result for the West Midlands region of which Birmingham is a part (+6%, CI −12% to +28%).

Our study used multilevel modelling on weekly RTC counts based on the 132 MSOAs in the city at level 2, with the predictor as the number of changed lights. In contrast, LANTERNS used conditional Poisson modelling on monthly (presumably calendar months) RTC counts based on road segments in multiple local authority areas, with the predictor as binary (unchanged or changed). The number of road segments involved in each analysis is not given. MSOAs in our study city varied between 0.53 and 13.13 km^2^ in area, contained multiple road sections and approx. 500–2000 lamps. In comparison, the LANTERNS analysis used individual road sections containing fewer lamps and which varied in length. Future analyses could benefit from more uniform analysis units, and additional reflection on the spatial scale of the analysis unit that best represents how driver behaviour is influenced by lighting quality.

There are also differences between these approaches relating to how darkness and daylight collisions were treated and how temporal trends were modelled. We examined weekly 24 hours, darkness, daylight and daylight-adjusted darkness RTC rates separately. LANTERNS only used the effect of the lighting change on the night collision rate adjusted for the daytime count^14^ (p25). Adjustment for the daytime count assumes that the lighting change only affects driver behaviour at night, an assumption which we question, as do Beyer *et al*^11^: ‘the assumption that street lighting does not affect day-time behaviour could be incorrect’.

We used a time-exposure ‘offset’ term in the separate darkness and daylight models in order to account for the seasonal variation in terms of length of daylight and darkness. Our multilevel approach also used a polynomial in time, giving a smooth, long-term underlying trend. This seems more realistic than the use of categorical years, which could lead to an unrealistic ‘staircase’ trend with a potential consequent impact on the result. In addition, our approach allows random effects in the underlying trend, so that this trend can vary in different areas. We think it is better to have the daylight effect manifest, as this could perhaps indicate useful further information to elucidate what might be changing the RTC rate.

More broadly, studies of lighting and safety must continue to reflect on the most appropriate measures of treatment and response. As clearly illustrated by Beyer *et al*,^11^ studies tend to compare RTCs in lit versus unlit streets, or areas with old versus new lighting technology. However, additional metrics might be beneficial to account for the heterogeneity of lamp design and lighting from roadside buildings. Similarly, Beyer *et al*^11^ divide studies into those that consider all road traffic crashes, or subsets of those involving injuries or fatalities. Our choice to focus on injury RTCs was a practical decision based on data availability, public health and statistical power. Not all minor RTCs are reported or cause physical harm. At the other extreme, fatalities are rare, so datasets large in time and space are needed to detect any impacts of lighting.

### Implications for urban health, planning and governance

Despite considerable evidence for strong links between the built environment and public health, much greater integration is needed between these fields in practice.^18^ Our study of injury collisions and road lighting provides some evidence that large infrastructure projects may not achieve their expectations and highlights the role that detailed temporal data and appropriate statistical analysis should have in evidence-based decision-making.

It is argued that a major barrier to improving health outcomes lies in the governance processes within cities.^19^ Effective governance is notoriously difficult; the broad management of urban areas has been described as a type of *wicked problem*.^20^ Cities have a diverse range of environmental and social challenges,^21^ which, due to their complex nature,^22^ can be difficult to solve.^20^ Technological interventions are to be welcomed, yet as we have shown here, they may have unintended outcomes,^23 24^ presenting additional challenges for city planning and governance.^25^ While predicting the impacts of lighting changes is fraught with difficulties, the approach employed here illustrates that, given access to sufficient data, it should be possible to estimate how effective a relighting project has been. The key to bringing clarity to this issue is controlling for background trends in RTCs by taking advantage of the fact that the rates of lamp replacement vary in each part of the city. An outstanding concern is whether urban governance systems have the capacity and incentive to support experimentation, evaluation and adaptation^26^ as part of relighting projects.

**What is already known on this subject**

- Little is securely known about the impact of large-scale brightening and whitening of street lighting on road traffic collisions (RTCs). Previous studies often lack evidence that there is protection against publication bias, regression towards the mean or underlying temporal trends in collision risk. In Europe, reductions in collisions might be expected over the duration of a study, notwithstanding changes to the road environment. Additional challenges include data quality and choosing an appropriate study design. A recent large-scale study involving lighting changes on tens of thousands of kilometres of roads in 62 local authorities in England and Wales found no good evidence that any changes were associated with harm or benefit. The changes, as well as reducing lighting, included switching to white/LED light. However, there remain questions over the most appropriate statistical approach and choice of response variable (eg, 24 hour vs darkness vs daylight adjusted darkness RTC rate).

**What this study adds**

- Our study was designed to examine the effect of relighting just one large UK city, Birmingham, on reported injuries from RTCs using a dataset covering 2005–2013. It involved tens of thousands of lamps being changed to broad spectrum (white) light. We examined this city in high detail, sampling at the neighbourhood scale and using a ‘multilevel’ modelling process which is different from that of the recent large-scale study. In our study city, large increases in bright white lighting were associated with increases in collisions in darkness and daylight. In addition, when the darkness injury rate was adjusted by the daylight rate, no increase or decrease was detected. Our results highlight the need for lighting to be taken seriously as a public health issue, for claims of reduced traffic collisions within relighting proposals to be questioned and for an open debate about the most appropriate modelling technique and choice of collision response variables for related studies.

## References

[1] Hölker F, Moss T, Griefahn B, et al The dark side of light: a Transdisciplinary research agenda for light pollution policy. *Ecology and Society* 2010;15:13 10.5751/ES-03685-150413

[2] Ciriminna R, Albanese L, Meneguzzo F, et al Led street lighting: a looking ahead perspective. *Green* 2015;5:83–94. 10.1515/green-2015-0020

[3] Kyba CCM, Hänel A, Hölker F Redefining efficiency for outdoor lighting. *Energy Environ. Sci.* 2014;7:1806–9. 10.1039/C4EE00566J

[4] Leeds City Council *Outline business case for PFI credits to the department for transport*. Leeds, UK: Leeds City Council, 2004.

[5] Surrey County Council *Street lighting private finance initiative: final business case*. Surrey, UK: Surrey County Council, 2009.

[6] Croydon and Lewisham Boroughs *Croydon and Lewisham street lighting PFI: final business case*. London, UK: Croydon and Lewisham London Boroughs, 2011.

[7] WHO *Injuries and violence: the facts*. Injuries and violence: the facts: World Health Organization, 2010.

[8] Eurostat *Road safety statistics at regional level*. European Union, 2014.

[9] UK Government *Number of fatalities resulting from road accidents in Great Britain*. London: Department For Transport, 2014.

[10] WHO *Global status report on road safety 2015*. World Health Organization, 2015.

[11] Beyer FR, Pond P, Ker K Street lighting for preventing road traffic injuries. *Cochrane Database of Systematic Reviews* 2010. 10.1002/14651858.CD004728.pub2 PMC11743125 19160240

[12] UN *Report of the United Nations Secretary-General’s High-level Panel on Access to Medicines. Promoting innovation and access to health technologies*, 2016.

[13] Marchant P Why lighting claims might well be wrong. *International Journal of Sustainable Lighting* 2017;19:69–74. 10.26607/ijsl.v19i1.71

[14] Perkins C, Steinbach R, Tompson L, et al What is the effect of reduced street lighting on crime and road traffic injuries at night? A mixed-methods study. *Public Health Res* 2015;3:1–108. 10.3310/phr03110 26401542

[15] Browne W *Mcmc estimation in MLwiN*. Bristol, UK: Centre for Multilevel Modelling, University of Bristol, 2009.

[16] Rasbash J, Charlton C, Browne W, et al *MLwiN version 2.1*. Bristol, UK: Centre for Multilevel Modelling, University of Bristol, 2009.

[17] IBM *IBM SPSS statistics for windows, version 23.0*. Armonk, NY: IBM Corp, 2013.

[18] Corburn J Confronting the challenges in Reconnecting urban planning and public health. *Am J Public Health* 2004;94:541–6. 10.2105/AJPH.94.4.541 15053998 PMC1448291

[19] Dye C Health and urban living. *Science* 2008;319:766–9. 10.1126/science.1150198 18258905

[20] Grimm NB, Faeth SH, Golubiewski NE, et al Global change and the ecology of cities. *Science* 2008;319:756–60. 10.1126/science.1150195 18258902

[21] Bai X, Shi P, Liu Y Society: Realizing China’s urban DREAM. *Nature* 2014;509:158–60. 10.1038/509158a 24812683

[22] McPhearson T, Haase D, Kabisch N, et al Advancing understanding of the complex nature of urban systems. *Ecol Indic* 2016;70:566–73. 10.1016/j.ecolind.2016.03.054

[23] Checker M Wiped out by the “greenwave”: Environmental gentrification and the paradoxical politics of urban sustainability. *City Society* 2011;23:210–29. 10.1111/j.1548-744X.2011.01063.x

[24] Ogden LE Does green building come up short in considering biodiversity? *Bioscience* 2014;64:83–9.

[25] Meijer A, Bolívar MPR Governing the smart City: a review of the literature on smart urban governance. *Int Rev Adm Sci* 2016;82:392–408. 10.1177/0020852314564308

[26] Ahern J From fail-safe to safe-to-fail: sustainability and resilience in the new urban world. *Landsc Urban Plan* 2011;100:341–3. 10.1016/j.landurbplan.2011.02.021

