# Supplementary material for: Does changing to brighter road lighting improve road safety? Multilevel longitudinal analysis of road traffic collision frequency during the relighting of a UK city
Source: J Epidemiol Community Health. 2020 May 1;74(5):467–72. doi: 10.1136/jech-2019-212208 (PMC7307661; doi:10.1136/jech-2019-212208)
Supplement: Supplementary data [file jech-2019-212208s001.pdf]

The protocol for investigating the effect of new brighter road lighting on traffic accidents.

Drafted by Paul Marchant, Final Version (12), 21 Jan 2015

UK Cities are implementing re-lighting programmes in which bright street lamps such as Light Emitting Diode (LED) and High Pressure Sodium (HPS) lamps are introduced, often under Private Finance Initiative (PFI) schemes. These lamps replace dimmer ones, e.g. Low Pressure Sodium (LPS) and Mercury Vapour (MV). The aim of this research is to determine what effect this change has on the number of Road Traffic Accidents (RTAs), against a background of anticipated generally declining accident rates. A systematic review of RTAs and road lighting suggested that lighting is very effective for reducing accidents (Beyer and Ker 2010). However the review attracted and includes substantial criticism. (Matters in the critical comments appended to the Cochrane Review include the effects of possible publication bias and regression towards the mean.) It is the purpose of this work to see what has happened in one city which is changing its lighting, including such a PFI scheme.

The RTA data to be used is that for personal injury accidents reported to the Police. This data is known as STATS19 and can be obtained via the Administrative Data Liaison Service. www.adls.ac.uk/department-for-transport/stats19-road-accident-dataset/?detail.

RTAs occurring both in 'darkness' and outside of this period are selected, so that the full 24 hours covered. The RTAs are allocated to the geographical area, the Middle Super Output Area (MSOA), of occurrence.

For each of the MSOAs a time series is constructed of the number of RTAs occurring in each of the weeks for which lighting data is obtained. Lighting data is available prior to the time that the PFI was agreed as well as after.

The strategy for the research is to analyse the time series resulting from counts of RTAs in each MSOA as a multilevel model with the time points at level 1 and MSOA at level 2. The modelling approach is to fit a time trend for an appropriate function of the mean number of accidents per week for the MSOAs. The time trend will be modelled as a polynomial, the degree of which is to be determined, e.g. a quadratic in time. It is anticipated that there may be a seasonal effect which will be modelled with sinusoidal terms. The modelling is then to be extended to incorporate a measure of lighting as new bright lamps are introduced, with a potential improvement in model fit, because of any effect of lighting on RTA probability.

The approach, given in this protocol, shares some similarities with work on annual crime counts in London boroughs (Marchant 2011). This research was unable to detect a difference between those boroughs which were re-lit with new street lighting and those that were not. However the uncertainty in the result of this earlier work is considerable because there were only few time units (years) and only few level 2 units (boroughs). However even so its result is inconsistent with that of a systematic review of experimental work on street lighting and crime (Welsh & Farrington 2008), which claims a substantial (20%) effect reduction of crime through new lighting. It should be noted that the lighting and crime systematic review has no protection against the same criticisms made against the Beyer and Ker review, cited above.

In this current proposed research, the Poisson distribution for the weekly accident counts, the 'response' variable, will be used. (The weekly counts will be small). The Poisson is anticipated to be satisfactory. However the potential need to take over-dispersion into account will be investigated, as will the possible use of the alternative, Negative Binomial distribution.

The natural logarithm of the counts of the response, i.e. the number of RTAs in each week within the area, will be used (as this is the canonical link function for the Poisson).

An analysis which examines the full effect of introducing brighter lamps will take the total number of accidents occurring in the full 24 hour period in the MSOAs as the response.

The modelling will proceed from simple to more complex, initially just fitting a time trend with seasonality. Then the effect of the increasing number of bright street lamps in the area, which is the key interest, will be introduced. Here the 'degree' of lighting (involving the number of bright street lamps) will be split into two

parts: 1) the deviation of the amount of brighter lighting at that time point from its mean value over the period of study in that area, 2) the mean in each area over the period of study, centred on the grand mean for all areas.

The primary measure of lighting in the log link model will be log (number of bright lamps), log (L_{ij}).

L_{ij} = the number of bright lamps at time i, in area j

The form is:

$$\log(\mu) = \beta_0 + \beta_1 t + \beta_2 t^2 + \beta_c \text{Cos}2\pi t + \beta_s \text{Sin}2\pi t + \beta_3(\log(L_{ij}) - \langle\log(L_{ij})\rangle_j) + \beta_4(\langle\log(L_{ij})\rangle_j - \langle\langle\log(L_{ij})\rangle\rangle)$$

where <> denotes mean, t= time in years the midweek is from the winter solstice prior to the start of the series.

The β_0 term is the intercept coefficient which is expected to be random, as different areas will have different accident rates, $\beta_1 t + \beta_2 t^2$ represent the time trend and $\beta_c \text{Cos}2\pi t + \beta_s \text{Sin}2\pi t$ seasonality; with additional harmonic terms potentially needed.

The β_3 term represents the deviation of the log number of lamps from its mean <> in the area, giving the within-area effect of lighting change. This coefficient will enable the effect of changing lighting to be seen. The β_4 term represents between area term, the deviation of the mean log number of lamps in each area from the grand mean <<>> of the log number of lamps over all areas. The advantage of this centring of the between area term is that the intercept will give the value of the log mean accident rate per week for a typical area, rather than one with a zero mean log number of lamps.

For this model, exponentiating for μ, the mean rate of accidents, will give a power law for L_{ij}, the number of lamps at time i, in area j.

Examining just night time accidents (in 'darkness', codes 4-7 in STATS19), rather than the full 24 hours, introduces an additional complication, as the exposure will vary with the length of the darkness throughout a year. The exposure term for the darkness duration for the latitude of the city is derived using standard astronomical expressions (see Appendix). These calculate the interval between sunset and sunrise. This is then reduced by one hour, as darkness is defined as beginning 30 minutes after the Sun has set and ending 30 minutes before it rises. With the exposure established, the model will incorporate an 'offset' (the log exposure in the log link model) to place results on an 'even footing'. Sinusoidal terms for seasonality in the model are expected to be necessary (potentially with harmonics) because for example the 'rush-hours' will include darkness at some parts of the year and not at others.

As stated above, the full series will be utilised for both 24 hour & darkness only analyses; although the analyses will be repeated for just the final years when operating under the PFI regime. A secondary model will use L_{ij} directly, rather than its log, in the model which will lead to an exponential form, rather than a power law. The modelling will be performed with the MLwiN package.

Because the response variable is a count, Maximum Likelihood Estimation can give suspect results & it is preferred that Markov Chain Monte Carlo (MCMC) is used instead. Model goodness of fit can be judged by the Deviance Information Criterion (DIC); the Bayesian analogue of the Akaike Information Criterion. It will be this which will determine for example the degree of polynomial for the time trend and whether parameters need to be 'fixed' or 'random'.

Exploratory analysis for darkness-only RTAs from 2005 up to 2012 has been already done for testing purposes. The analysis for which this protocol is written will include RTA and lighting change data up to the end of 2013.

The models will be repeated using the Generalized Estimating Equation (GEE)s approach, available in SPSS, as opposed to the multilevel , i.e. Generalized Linear Mixed Model (GLMM), approach.

Sensitivity Analysis will be performed to see if changes to modelling assumptions alter results.

Any amendments to the protocol that are needed during the analysis will be recorded and pointed out.

References

Beyer FR and Ker K. (2010), Street lighting for preventing road traffic injuries, Cochrane Database of Systematic Reviews 2009, Issue 1. Art. No.: CD004728. DOI: 10.1002/14651858.CD004728.pub2.

Marchant PR (2011), Have new street lighting schemes reduced crime in London?" Radical Statistics, 104, pp39-48 - http://www.radstats.org.uk/no104/Marchant2_104.pdf

Welsh BC and Farrington DP (2008), Effects of improved street lighting on crime, Campbell Systematic Reviews DOI: 10.4073/csr.2008.13

Appendix: The Calculation of the Length of Darkness.

The Universal Time (UT) of the rise and set of an object

$= 0.9972 * (\propto + \lambda \mp \arccos\ (-tan\phi\ tan\ \delta) - GST at\ UT\ 0^h)$

Right Ascension = α, Declination = δ, Longitude (West) = λ, Latitude (North) = ϕ, GST = Green Sidereal Time.

0.9972 = the sidereal drift with respect to Universal Time

The minus sign is for 'rise', while 'set' takes the plus

Therefore the difference, Rise-Set = - 2 x 0.9972 $\arccos\ (-tan\ \phi\ tan\ \delta)$

(Note, for rising and setting to occur the argument of arc cosine must reside between -1 and +1, as it will for the Sun, outside of the Polar Regions.)

To account for refraction in the Earth's atmosphere & the size of the Solar disc an additional term $= {\cos z}/{\cos\delta\cos\phi}$ needs to be added to $-tan\ \phi\ tan\ \delta$, with z= 90.833 degrees

The Sun's declination δ = $-\psi\cos(\frac{2\pi t}{T})$

ψ = obliquity of the Earth's spin axis to the ecliptic 23.439 degrees

T = the length of the tropical year = 365.242 days, t = the number of days from the Winter Solstice preceding the time series, evaluated at the middle of each of the weeks of the series.

In the UK, 'darkness' is defined as starting ½ hour after sunset & ending ½ before sunrise, therefore the darkness period is one hour less than sunset-sunrise interval.

Reference

The Handbook of the British Astronomical Association: An Explanatory Supplement

http://britastro.org/computing/pdf/BAA_Handbook_Explanatory_Supplement_1988.pdf

AMMENDMENT_1 24 Feb 2015

In the protocol the (canonical) log link for the mean of the RTA count (per week) has been used in the linear models. The log link is a favoured link function for counts, as on exponentiating back to the count scale, a non-zero number for the mean is guaranteed.

In the protocol the favoured, primary, model is given as:

Model1: $\log(\mu_{ij}) =$

$$\beta_0 + \beta_1 t + \beta_2 t^2 + \beta_c \mathrm{Cos}2\pi t + \beta_s \mathrm{Sin}2\pi t + \beta_3(\log(L_{ij}) - <\log(L_{ij})>_j) + \beta_4(<\log(L_{ij})>_j - < <\log(L_{ij})>>)$$

On transforming; the $\beta_3(\log(L_{ij}) - <\log(L_{ij})>_j)$ term becomes the factor $(L_{ij} / <(L_{ij})>_j)$ ^ β_3 , that is a power function of the number of bright lamps L_{ij} in the product of terms for μ_{ij}.

On the other hand, the secondary model, the linear model with the raw number of bright lamps L_{ij} , was less favoured, because of the log link, on transforming, would lead to an exponential factor for the number of lamps in the product of terms for μ_{ij}

Model2: $\log(\mu) = \beta_0 + \beta_1 t + \beta_2 t^2 + \beta_c \mathrm{Cos}2\pi t + \beta_s \mathrm{Sin}2\pi t + \beta_3(L_{ij} - <L_{ij}>_j) + \beta_4(<L_{ij}>_j - < <L_{ij}>>)$

[On transforming, the $\beta_3(L_{ij} - <L_{ij}>_j)$ term becomes the factor $\exp(\beta_3(L_{ij} - <L_{ij}>_j))$]

However it is now realised, with regret, that the power function of model1 is problematic, because comparing the effect on the mean at the end i =T to that at the beginning i =1 gives for an area j

$\mu_T / \mu_1 = (L_T / L_1)$ ^ β_3

So if an area has a small change, say from 10 to 20 bright lamps, the effect will be the same as for a bigger change from 100 to 200. This seems an unreasonable result. A perhaps more realistic situation would be obtained by having additional β_3' and β_4' terms with logarithms of l_{ij} , the number of dim lamps still extant at time i, that are being replaced by brighter ones. Note $l_{ij} + L_{ij} = N_j$ the total number of lamps in area j, which stays constant.

However, on reflection, Model 2 which generates the exponential factor, $\exp(\beta_3(L_{ij} - <L_{ij}>_j))$, seems acceptable as comparing the effect at the end to the beginning gives:

$\mu_T / \mu_1 = \exp(\beta_3(L_T - L_1))$

Therefore the ratio of effects depends on the absolute difference in the number of bright lamps at the end to the start. Also if $\beta_3(L_T - L_1) << 1$ the effect is $1 + \beta_3(L_T - L_1))$ which seems reasonable. (Note in this model it would be futile to include the number of dim lamps as this is perfectly negatively correlated with the number of bright lamps through $l_{ij} + L_{ij} = N_j$)

Therefore although the plan is to present both models, as was the case before, in this amendment the focus is on Model2, i.e. that containing a term with the raw number of lamps in the linear model.

(It might be instructive to construct a multilevel model using the identity link function rather than the log link, with the raw number of bright lamps; however the package MLwiN does not allow this.)
