# Supplementary material for: Does changing to brighter road lighting improve road safety? Multilevel longitudinal analysis of road traffic collision frequency during the relighting of a UK city
Source: J Epidemiol Community Health. 2020 May 1;74(5):467–72. doi: 10.1136/jech-2019-212208 (PMC7307661; doi:10.1136/jech-2019-212208)
Supplement: Supplementary data [file jech-2019-212208s002.pdf]

Methods - supplementary information

The following additional information is supplied to complement the core methods section of the main manuscript:

1) Street lamp data - additional information ... 1
2) Road Traffic Collisions - additional information ... 4
3) Multilevel modelling - additional information ... 8
4) SPSS syntax for calculating the number of days the Midweek is from the Winter Solstice (21 Dec 2004) ... 12
5) The SPSS syntax for calculating the Darkness time-exposure ... 13
6) References ... 14

1) Street lamp data - additional information

In 2010, the management of the city of Birmingham's street lighting was contracted out to the private sector, under a 25-year Private Finance Initiative scheme. The programme of works included an intense period of investment during the first 5 years, replacing 40,000 street lamps; intended outcomes of this shift to brighter white lighting included savings in energy and carbon emissions and an improvement in road safety [1].

Marchant P, et al. J Epidemiol Community Health 2020;0:1–6. doi: 10.1136/jech-2019-212208

The Low-Pressure Sodium (LPS) and Mercury Vapour (MV) lamps present in the city typically provided low levels of illuminance (2-5 lux in the horizontal plane at ground level), compared to a brighter 15-30 lux for the other (broad spectrum) lamp types.

Errors in this dataset were identified and corrected. ‘Ghost lamps’ are lamps which had previously been in place, were subsequently replaced, yet the associated record had not been deleted from the database. Typically, these had the same exact grid reference values as the lamp that had replaced them. The result in this example would be a single location with two lamp records – one for the old lamp and one for its replacement. 1501 records for ghost lamps were identified and removed from the dataset. 52 lamps had no associated spatial data and were therefore removed. Also 10 duplicate records were identified and removed. The final street lamp total for the city was 92,947. We restricted our analysis to lamps installed between Monday 03 Jan 2005 and Sunday 29 Dec 2013, to match the period 2005-2013 over which road traffic accident data were obtained for the city, and ensure whole weeks were used.

Given that the aim of the study was to assess the association between a shift towards brighter street lighting and changes in road accidents, we need a clear definition of what constituted a bright lamp. A distinction was made between the Low-Pressure Sodium (LPS) and Mercury Vapour (MV) street lamps found in the city, which were typically poorly shielded and had low luminous flux, and other lamps such as High-Pressure Sodium (HPS), Metal Halide (MH), Florescent, Tungsten and LED, which were much brighter and whose emissions covered a broad part of the visible light spectrum. LPS and MV were therefore classified as *dull lamps*, whilst the others were classified as *bright lamps*

Marchant P, *et al. J Epidemiol Community Health* 2020;0:1–6. doi: 10.1136/jech-2019-212208

Whilst a street lamp inventory was available for 2014, no equivalent inventory was available for the start of 2005. Our analysis therefore assumes that all new bright lamps installed from the start of 2005 replaced dull LPS or MV lamps.

The street lamp database was converted to a GIS point shapefile, using ArcGIS 10.2 (ESRI, Redlands, California). The 'date of installation' field was used to generate a second date field that represented the week since 03/01/05 that the lamp installation took place, using the 'DateDiff' function within field calculator. The data were overlaid with a polygon shapefile representing the Middle Layer Super Output Areas (MSOA) for the city, for the 2011 UK census. A MSOA is a geographical unit used in the UK to collect neighbourhood statistics; each has a minimum of 5000 residents (average = 7200). For Birmingham the mean number of residents = 8,139, median = 7,868, min = 5,277 and max = 14,788. For Birmingham, the mean MSOA area = 2.03 km^2, median = 1.70, min = 0.53 and max = 13.13. Each lamp record was assigned its corresponding MSOA code using the 'isectpntpoly' tool within GME 0.7.4.0 [2] and R 3.3.1 [3]. The data were then exported as an excel file and a summary generated using a pivot table, to count the number of bright lamps installed each week within each MSOA.

The increase in the number of bright lamps within the 132 MSOAs over the analysis time period can be expressed as either the arithmetic difference of the number of bright lamps in the MSOA at the end minus that at the start, or alternatively as the ratio of the final number of bright lamps divided by the initial number in the MSOA. The descriptive statistics of these two numerical measures of change of road lighting in the MSOAs from 03 Jan 2005 to 29 Dec 2013 are given in Table 1 below. Additional information on the minimum,

maximum and mean number of bright lamps/MSOA at the start & end of the time series is given in Table 2.

Table 1. The change in the number of bright lamps in the MSOAs over the analysis time period (both as a difference and as a ratio).

Measure of change	N	Minimum	Maximum	Mean	Std. Deviation
Number Increase in Bright Lamps i.e. Max-Min	132	9	680	273.66	149.77
Ratio of final number to initial number of bright lamps	132	1.01	22.38	2.65	2.47

Table 2. The descriptive statistics for the number of bright lamps at the start & end of the time series in the 132 MSOAs

	Minimum	Maximum	Mean	Std. Deviation
N Bright Lamps at Start	29	1590	334.05	233.681
N Bright Lamps at End	254	1956	607.70	244.665
N all Lamps (bright & dim)	325	2149	704.18	265.651

2) Road Traffic Collisions - additional information

The following codes for light conditions were provided as part of the STATS19 data:

1. Daylight

4. Darkness: street lights present and lit

5. Darkness: street lights present but unlit

6. Darkness: no street lighting

7. Darkness: street lighting unknown

(Codes 2 and 3 are not used)

Each collision record was associated with fields representing their spatial location (Ordnance Survey National Grid reference), allowing collision data to be converted to a point shapefile within the GIS. There is evidence that the STATS19 data includes misclassification errors where the time given for the RTC is not consistent with the lighting code (the claim for darkness or daylight). This was found using an algorithm which uses the date, latitude and longitude given for the RTC to calculate the sunrise and sunset times and hence the onset and end of darkness; by adding or subtracting 30 minutes. Some inconsistencies are extreme e.g. with some records claiming an RTC to be in darkness occurring around noon. It was not possible to correct these misclassifications, as it cannot be determined whether it is the lighting code or the collision time that are incorrect. However, the degree of misclassification was <5% overall. This was mostly due to reporting the collision as occurring in darkness when the time suggests that it occurred in official daylight, possibly because the street lights were in operation beyond the official darkness times. MSOA weekly summaries for (daylight and darkness) collisions were generated in the same way given for generating lamp summaries (see above section). The number of daylight collisions was added to the number of darkness collisions to give the round the clock (24h) rate occurring each week in each MSOA, the primary outcome variable for the study.

The frequency of RTCs per week, in the 61908 (= 132x469) MSOA x week combinations, is given in Table 3.

Table 3. Frequency of RTCs/week for the analysis time period.

Number of RTCs per week	Occurrences in the full 24h period	Occurrences in period of darkness	Occurrences in period of daylight
0	41007	54779	45857
1	15388	6287	12681
2	4068	739	2686
3	1070	93	544
4	275	9	112
5	72	1	20
6	23	0	7
7	5	0	1
Sum	61908	61908	61908

The distribution of the mean RTC rates follow Log-Normal distributions closely (as can be seen from the Q-Q plots in Fig 1 below). This is convenient for the count modelling using the log link function, as the level-2 residuals can be modelled as samples from a Normal distribution.

CONTINUED BELOW

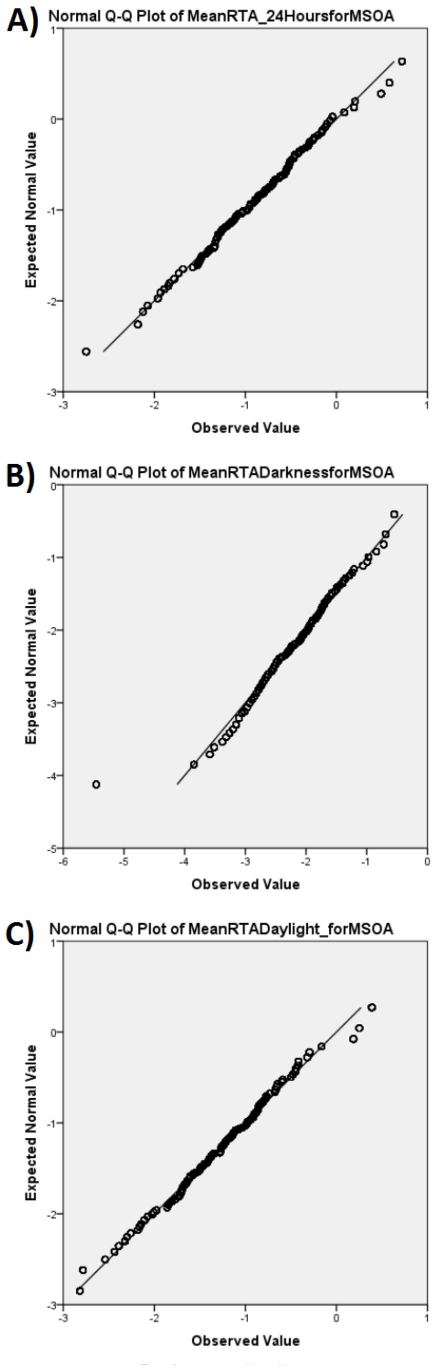

Fig 1. Normal Q-Q plot for A) mean of all MSOA RTAs, B) Darkness RTAs only and C) Daytime RTAs only.

3) Multilevel modelling - additional information

The measure of lighting used in the primary analysis was the number of new bright lamps operating, each week but a secondary analysis was undertaken using the log (number of bright lamps) (as specified in the protocol, S3).

We ran a full 24h model using Markov Chain Monte Carlo (MCMC) estimation in the multilevel analysis. This allowed the Deviance Information Criterion to be used for model selection. The modelling was then repeated with some modification for darkness and daylight RTCs. We then repeated this approach just using the last 3 years of data, and again using the full time series but with a Generalized Estimating Equation approach instead of Multilevel Modelling.

The progress of the relighting project was denoted as the change of the number of bright lamps within each area from its mean number. The models also included a second lighting term for the difference of a MSOA's mean number of bright lamps from the (grand) mean number across all MSOAs. The two terms for the build-up of bright lighting were thus centred. (An advantage of this centring is a more straightforward interpretation of such as intercepts.)

The protocol prescribed that the modelled time trend would include harmonic terms to account for seasonal variation throughout the year, as well as the long term, secular polynomial terms (with a degree to be determined). The model in the protocol is:

$\log(\mu_{ij}) = \beta_0 + \beta_1 t + \beta_2 t^2 + ... + \beta_c \mathrm{Cos} 2\pi t + \beta_s \mathrm{Sin} 2\pi t + \beta_W(L_{ij} - <L_{ij}>_j) + \beta_B(<L_{ij}>_j - <<L_{ij}>>)$

The terms, $\beta_c \mathrm{Cos} 2\pi t + \beta_s \mathrm{Sin} 2\pi t$, accommodate seasonality; with additional higher harmonic terms potentially needed to adequately represent seasonality.

Where $<>_j$ denotes the mean with respect to week i in area j, $<<>>$ the mean of the area means and t = the time that the midweek is from the winter solstice prior to the start of the series (21 Dec. 2004).

The β_0 term, the intercept coefficient was modelled as a random term because different areas will be differentially busy; $\beta_1 t + \beta_2 t^2 + \cdots$ represents the underlying secular time trend, a polynomial with a degree to be determined. Polynomial coefficients, e.g. β_1, might also be expected to be random because of different temporal changes between different MSOAs. The β_W term represents the effect of the deviation of the number of bright lamps, L_{ij} , from its mean <>, over the time series duration, in the area, giving the within-area effect of lighting change. This coefficient enables the effect of changing lighting to be seen. The β_B term is the between-area term; the effect of the deviation of the mean number of bright lamps in an area, over the series, from the mean of the MSOA means.

The form of the model actually used was:

$\log(\mu_{ij}) =$

$\beta_0 + \beta_1 t + \beta_2 t^2 + \cdots + \beta_{Mk} \mathrm{Month}_k + \beta_{Hl} \mathrm{PubHol}_l + \beta_W(L_{ij} - <L_{ij}>_j) + \beta_B(<L_{ij}>_j - <<L_{ij}>>)$

The harmonic terms were replaced with indicator variables for the month of the year. This was because it was found that the number of harmonic terms that would be needed in the model, for a good fit to the data, was more than the number of months in a year. Changing to months has the advantage of making the modelled results more readily interpretable. Additionally, seven indicator variables for weeks that included each of the annual public holidays were added, in order to reduce background effects on the RTC rate, as public

holiday weeks tend to have lower rates. In practice though, both seasonality and public holidays only had a miniscule effect on the key estimate, that of the impact of the number of bright lamps in an area, β_W.

The β_{Mk} term represents the effect of the k = 1 to 11 Month indicator variables (reference = January) and β_{Hl} that of the 7 public holiday weeks per year l = 1 to 7 (reference = weeks which are not public holiday weeks). The latter being two ‘deviations from protocol’ made during the analysis

In order to prepare for modelling, we identified the midweek date (three days on from the Monday) and calculated the number of days this was from the winter solstice that preceded the start of the time series, (December 21, 2004) (The winter solstice has minimum daylight & maximum darkness). The SPSS syntax for this process is given in section 4 below. This then allowed calculation of the length of darkness, in hours, for the latitude of the city, for each of the weeks in the data (for SPSS syntax see section 5 below). This in turn allowed creating an ‘offset’ for modelling RTCs occurring in darkness, and also for those occurring in daylight.

The time in days of the midweek from the winter solstice, preceding the series, was converted to years (t) by dividing by 365.242, the number of days in a tropical year, in order to have a more convenient unit of time. This was originally to allow the harmonic terms for seasonality to be easily created of the form $\text{Cos}2\pi nt$ & $\text{Sin}2\pi nt$, with n=1, 2,⋯ as required by the original protocol. However, as stated the deviation from protocol was made whereby the harmonic method of analysis, to address seasonality, was replaced by the direct approach of using the month of the year with reference category of January.

The daily hours of darkness and daylight exhibit seasonality, that is, darkness is long, and daylight is short in winter. The reverse situation applies in summer. Therefore, one expects the number of darkness RTCs to be higher in winter and those in daylight to be higher in the summer (The time-exposure is of course constant for the RTCs occurring over the full 24 hours). To compensate for the varying time-exposures in both darkness and daylight RTCs, the models for these counts of RTCs included an ‘offset’ for the appropriate fraction of 24 hours, thereby giving an effective (log) rate scaled to 24 hours. This offset was calculated in SPSS, the syntax for Darkness length (from which Daylight length can be calculated by subtraction) can be found in section 5 below. Using an offset had the beneficial effect of handling the otherwise strong seasonal effect on the RTC counts associated with the varying time-exposure. Some remaining seasonality in the observed collision rate might be expected as the number of vehicles exposed to risk will be variable over a year for both darkness and daylight because for instance the rush-hours will occur in darkness in winter but not in summer.

We ultimately cast the time variable into a unit of a decade in order that the values of coefficients of the temporal polynomial were of a convenient size, neither too big nor too small). The amount of new lighting was put into units of 100 lamps, again to obtain conveniently sized coefficient values.

The multilevel modelling was repeated on just the last 3 years of the time series (Jan 2011 to Dec 2013). This was a period when the Private Finance Initiative programme was fully underway, when a rapid growth in the number of bright lamps occurred.

In order to check that there were no obvious errors in the above approach, Generalized Estimating Equation (GEE) models were also run on the full time series 2005 - 2013 in SPSS, using the same model terms for 24h RTC rates and the darkness and daylight rates.

Given that the data are well represented by the fitted models, to investigate the association of new lamps with RTCs reported as occurring in darkness compared to those occurring daylight, we note that the daylight model can be subtracted from the darkness one. This gives $\log(\mu_{dark}) - \log(\mu_{daylight}) = \log(\mu_{dark} / \mu_{daylight})$, that is the log of the ratio of the mean weekly RTCs in darkness to the mean weekly RTCs in daylight. This is equal to the difference of the linear combinations predicting each log RTC mean.

In addition to differencing the coefficients from the individual darkness and daylight RTC-rate models to obtain estimates of the daylight-adjusted effects, a variety of models were also run. These included 1) bivariate 2) binomial 3) incorporating a binary (0,1) darkness indicator regressor using a ‘stacked’ form of the data, i.e. separate rows for darkness rate and daylight rate, giving twice as many cases.

While it is possible to adjust the darkness RTC rate by the daylight rate, we felt that in the interests of transparency, it was preferable to have the daylight RTC rate explicitly reported (see Discussion in main manuscript).

4) SPSS syntax for calculating the number of days the Midweek is from the Winter Solstice (21 Dec 2004)

```
COMMENT The Dates of the start of each week.
COMPUTE WeekMonStartDMY =DATE.DMY(03,01,2005) + (WeekNum-1)*3600 * 24 * 7.
EXECUTE.
COMMENT Number of days the Midweek is from the Winter Solstice previous to the start of the series.
COMPUTE DaysMidWeekFromWSolstice=(DATE.DMY(06,01,2005)
-DATE.DMY(21,12,2004))/3600/24 +(WeekNum-1)*7.
```

EXECUTE.

5) The SPSS syntax for calculating the Darkness time-exposure

COMMENT Generate Pi then Degree to Radian Conversion.

COMPUTE #PI = 4* ARTAN(1.0).

COMPUTE #DegToRad = #PI /180 .

COMMENT Put Latitude in Radians.

COMPUTE #LatitudeRad = 52.5 * #DegToRad .

COMMENT Generate Sun's Declination.

COMPUTE #SunDeclDeg = -23.439*COS(2 *#PI* DaysMidWeekFromWSolstice / 365.242).

COMPUTE #SunDeclRad = #SunDeclDeg * #DegToRad.

COMPUTE #TanLat=SIN(#LatitudeRad)/COS(#LatitudeRad).

COMPUTE #TanSunDecl = SIN(#SunDeclRad)/COS(#SunDeclRad).

COMMENT Calc Cosine Hour Angle Of Sun With Final Correction term for both Re & Disc size .

COMPUTE #CosHourAng= -#TanLat* #TanSunDecl

+COS(90.83333333*#DegToRad)/COS(#SunDeclRad)/COS(#LatitudeRad).

COMMENT SPSS Does NOT have ARCOS so need to use the following to give HourAng 0 to PI.

COMPUTE #HourAng= #PI/2 - ARSIN(#CosHourAng).

COMMENT Calculate NightLength -2* 0.9972* HourAng is the angle Rise and Set.

COMPUTE #NightLength =24*(1- 2* 0.9972* #HourAng /(2 * #PI)).

COMMENT Darkness starts 1/2hr after sunset and ends 1/2hr before sunrise.

COMPUTE DarkLength = #NightLength-1.

EXECUTE.

Note the Daylight time-exposure is the complement of Darkness time-exposure

6) References

1 AMEY. Case Study. Birmingham Highways Maintenance and Management Service. UK: AMEY plc 2012:12.

2 Beyer HL. Geospatial Modelling Environment Version 0.7.4.0 (software). http://www.spatialecology.com/gme. 2015.

3 R Core Team. R: A language and environment for statistical computing Vienna, Austria: R Foundation for Statistical Computing 2015.
