# Supplementary figures and images for: Does changing to brighter road lighting improve road safety? Multilevel longitudinal analysis of road traffic collision frequency during the relighting of a UK city
Source: J Epidemiol Community Health. 2020 May 1;74(5):467–72. doi: 10.1136/jech-2019-212208 (PMC7307661; doi:10.1136/jech-2019-212208)

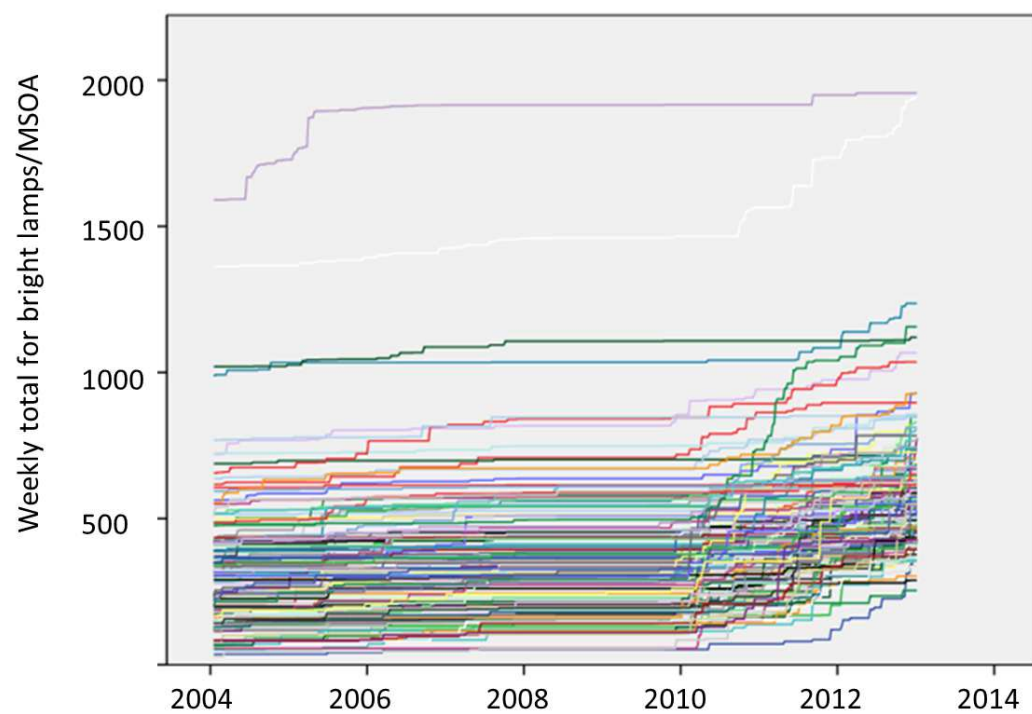

Figure S3. The build-up of bright lights within each MSAO over time (totals at weekly intervals).

Supplement: Supplementary data [file jech-2019-212208s003.pdf]
