# Supplementary material for: Does changing to brighter road lighting improve road safety? Multilevel longitudinal analysis of road traffic collision frequency during the relighting of a UK city
Source: J Epidemiol Community Health. 2020 May 1;74(5):467–72. doi: 10.1136/jech-2019-212208 (PMC7307661; doi:10.1136/jech-2019-212208)
Supplement: Supplementary data [file jech-2019-212208s004.pdf]

Results - supplementary information

1. Temporal model components ... 1
2. The secondary analysis (using Log bright lamps) ... 2
3. Three-year period of intense lamp replacement ... 4
4. The Generalized Estimating Equation alternative approach ... 4
5. Model validation ... 5
6. Adjusting for daylight collision rate ... 6
7. Additional interaction terms and an interruption variable ... 7

1. Temporal model components

A key issue in clarifying any effects of lighting change is separating the underlying time-variation of RTC rate from the change in RTC rate associated with the numbers of installed bright lamps. Our multilevel modelling indicated that a polynomial in time of the 4th degree was adequate for all of 24-hour, darkness and daylight RTC models when using the full time series from 03 Jan 2005 up to 29 Dec 2013. As the degree was increased to the 4th power, the within area coefficient β_W of the number of bright lamps decreased, but extending to the 5th degree maintained a similar positive value for the within area effect. A consequence of extending the degree of the polynomial to 4th power was that the Markov Chain Monte Carlo MCMC run length had to be increased, mostly to 2 million, in order to ensure that the effective sample size in the estimation of the parameters for the time terms exceeded 100.

The form of the model used is:

$$\log(\mu_{ij}) = \beta_0 + \beta_1 t + \beta_2 t^2 + \ldots + \beta_{Mk}\text{Month}_k + \beta_{Hl}\text{PubHol}_l + \beta_W(L_{ij} - \langle L_{ij}\rangle_j) + \beta_B(\langle L_{ij}\rangle_j - \langle\langle L_{ij}\rangle\rangle)$$

All the time-polynomials in the optimum fitted models had a random constant term, as expected. This was the intercept term and the random effect reflects the varying level of RTCs in different MSOAs. The other random time terms in the polynomial reflected the variation of underlying RTC-trends between areas. The final 24h and daylight models had the linear and quadratic terms random but the cubic and quartic terms fixed. The darkness polynomial had all its terms fixed apart from the intercept.

2. The secondary analysis (using Log bright lamps)

When the logarithm of the number of bright lamps was used instead of the number of bright lamps, the form was:

$\log(\mu) =$

$\beta_0 + \beta_1 t + \beta_2 t^2 + ... + \beta_{Mk}Month_k + \beta_{Hl}PubHol_l + \beta_w(\log(L_{ij}) - <\log(L_{ij})>_j) + \beta_B(<\log(L_{ij})>_j - <<\log(L_{ij})>>)$

The polynomials in time, when using the logarithm of the number of lamps, were taken to be of the same form as those in the primary analysis, which used the number of lamps. (All the coefficients in the log L_{ij} model were newly estimated during the fitting). The within area estimate on exponentiation to obtain the RTC rate, rather than its log, gives a power-law relationship for the lamp effects, as noted in the protocol (S1 Appendix). That is; the ratio of the rates, final to initial, adjusted for the underlying temporal variation for any area was the ratio of the number of lamps, final to initial, raised to the power of the within area coefficient.

That is $\mu_{lj} / \mu_{1j} = (L_{lj} / L_{1j})^{\beta W}$

For the 24hour rate, the within-area coefficient was 0.1193 (0.0474) and the between area effect was 0.4942 (0.0810). Again, the within area lamp effect is of principal interest, giving as it does the expected

effect on RTCs arising from the change of the lighting in any area. For the darkness rate, the within area effect was 0.1206 (0.0726) and the between 0.5617(0.0938). For the daylight rate, the within area effect was estimated to be 0.1445 (0.0531) and the between 0.4806 (0.0781). The positive between area effect is unsurprising, as in the primary analysis, because areas with greater average numbers of bright lamps installed might be expected to have more traffic.

These results are summarised in Table 1 below, which also displays the increases in the RTC rate for the mean ratio of the number of bright lamps at the end of the series to that at the start (2.66). One can clearly see that the results for areas with typical lighting increases have similar confidence intervals irrespective of whether it is the number of lamps or its log which is used in the modelling

Table 1: The within area effects of bright lamps for 24-hour, Darkness and Daylight, using the logarithm of the number of bright lamps. The percentage increases are the effect on the number of RTCs for the mean ratio of the number of bright lamps, final to initial (=2.66).

	Within MSOA coefficient, for 100 bright lamps added.	Within MSOA SE	Increase in RTCs for a 2.66 ratio of lamps	LCL	UCL
24-hour	0.1193	0.0474	12%	3%	23%
Darkness	0.1206	0.0726	13%	-2%	29%
Daylight	0.1445	0.0531	15%	4%	28%

3. Three-year period of intense lamp replacement

The findings of this modelling were consistent with the full time series: a within area coefficient of 0.0111 (0.0249) with the between area effect of 0.0836 (0.0189). The within-area effect confidence interval was considerably wider (by > 50%) due to the reduced sample. Only a quadratic temporal model was required, presumably because of the short length of the time series.

4. The Generalized Estimating Equation alternative approach

The results of the GEE modelling approach were similar to those of the equivalent multilevel analyses, but the point estimates of the within MSOA coefficients of the effect of the number of lamps (Table 2) were a little larger and the confidence intervals a little wider than found in the primary analysis

Table 2. Comparing the within area effects of bright lamps for 24-hour, Darkness and Daylight using the number of bright lamps, using GEEs, with independent correlation and robust SEs. The percentage increases on the number of RTCs are the effect for 274 (the mean increase) in the number of bright lamps on RTCs.

	Within MSOA coefficient, for 100 bright lamps added	Within MSOA SE	Median RTC increase for 274 lamps replaced	LCL	UCL
24-hour	0.0561	0. 0200	17%	5%	30%
Darkness	0.0695	0.0250	21%	6%	38%
Daylight	0.0504	0.0223	15%	2%	29%

5. Model validation

The Pearson residuals for the Poisson analyses on the primary outcome (24hour rate) model seemed satisfactory as when grouped into ten bands of expected count, as the means were all approximately zero with standard deviations close to one (Table 3). Alternatives to the Poisson probability distribution were investigated for the full 24h data, by switching to over-dispersed Poisson and Negative Binomial responses in the MLwiN model. The over-dispersion parameter was seen to be effectively one and the extra term in the Negative Binomial was effectively zero. Therefore, no need for increased distributional complexity was evident.

Serial correlation might have been detected in the above model validation steps, if it had been present. However, to investigate this directly, an autoregressive model was run which showed that the coefficient of the autoregressive term was not statistically significantly different from zero and that the effect of lighting was unchanged. It might be expected that autoregression would not be a problem in our situation because of the geographical scale of the areas (i.e. MSOAs).

Table 3. Means and Standard Deviations of Pearson residuals of the decile bands of expected RTA count

Decile band	Mean	Standard Error	StdDev
1	-.0094	.01248	0.98218
2	-.0290	.01228	0.96616
3	.0107	.01298	1.02105
4	.0056	.01278	1.00585
5	.0190	.01296	1.01997
6	-.0181	.01258	0.98975
7	.0081	.01274	1.00257
8	-.0018	.01275	1.00289
9	.0092	.01287	1.01294
10	.0106	.01275	1.00308
Total	.0005	.00402	1.00080

6. Adjusting for daylight collision rate

The log ratio of darkness to daylight RTCs with new lamps is given by the difference of the 2 within area coefficients for lamps. That is, the lamp effect for log (μ_{dark} / $\mu_{daylight}$) = 0.0549 – 0.0395 = 0.0154. The associated standard error is given by $(0.0242^2 + 0.0193^2)^{0.5}$ = 0.0310 on the assumption of statistical independence. Thus, the standard error of this log ratio of means is larger than its point estimate and therefore indicates no statistically significant difference from zero. The point estimate of change due to brightening in an area receiving the average number of brighter lamps is 4% in a 95% CI (-12%, +23%).

In order to compare with the darkness minus daylight differencing method, for obtaining the effect of new lighting adjusted by the daylight RTC rate, other approaches were also performed, although not prescribed in the protocol. These modelled jointly the darkness and daylight RTC rates; one used a logistic binomial approach. A reassuring consequence of using another, an equivalent bivariate model, was that it found the level 1 correlation of weekly darkness and daylight RTC events to be = -0.0027 (0.0040). That is the correlation is indistinguishable from zero, thereby justifying the assumption of independence implicit in the differencing method. All approaches gave consistent values of the point estimates, within the (consistent) values of standard error. All estimates of the effect of lighting on the ratio of darkness to daylight RTCs were not statistically significantly different from zero and were close to each other. The difference method for an area receiving the average number of new lamps gives an increase of 4% in a CI (-12%, +23%), as stated above, whilst the bivariate model gives 6% (-9%, +25%) and the binomial model 5% (-10%, +22%).

Marchant P, *et al. J Epidemiol Community Health* 2020;0:1–6. doi: 10.1136/jech-2019-212208

7. Additional interaction terms and an interruption variable

Two additions to the final model were explored: adding an interaction term between lighting and time, and a dummy variable to denote the intense relighting period from January 2011. Ultimately these were not included in the final model as the estimate of their coefficients were small in magnitude and dwarfed by their respective standard errors.

Table 4. Results of including interaction terms and a dummy variable for the interruption to the time series

	**24h dataset**		**Dark**		**Day**	
**Analysis**	Coefficient	SE	Coefficient	SE	Coefficient	SE
MCMC	0.0366	0.0159	0.0549	0.0242	0.0395	0.0193
MLE IGLS	0.0363	0.0157	0.0551	0.0243	0.0383	0.0175
**Including Interaction t**						
L main effect	0.0411	0.0399	0.0405	0.0706	0.0530	0.0440
L.t interaction	-0.0078	0.0591	0.0229	0.1043	-0.0239	0.0657
**Including Interaction t^2**						
L main effect	0.0554	0.0330	0.0720	0.0580	0.0594	0.0368
L.t^2 interaction	-0.0405	0.0612	-0.0343	0.1067	-0.0452	0.0692
**Change of lighting slope Pre-Post Jan2011**						
Before	0.0372	0.0331	0.0409	0.0583	0.0532	0.0367
Increment after	-0.0013	0.0431	0.0207	0.0772	-0.0222	0.0481

The top two rows show results (coefficient estimates and their standard errors) for the models as in the paper. It shows that Max Likelihood Estimation (which takes seconds) results are very close to the

Marchant P, *et al. J Epidemiol Community Health* 2020;0:1–6. doi: 10.1136/jech-2019-212208

MCMC ones (which take many hours or even a day). So, all subsequent modification to the models is done with MLE.

We see that the estimates of all the interaction coefficients are smaller than their SEs, indicating these are not needed. Similarly, the coefficients for the increment to the slope in the period 2011-2013 are smaller than their SEs showing that the change of slope is undetectable, indicating overfitting.

Marchant P, *et al. J Epidemiol Community Health* 2020;0:1–6. doi: 10.1136/jech-2019-212208
