# Supplementary material for: Does changing to brighter road lighting improve road safety? Multilevel longitudinal analysis of road traffic collision frequency during the relighting of a UK city
Source: J Epidemiol Community Health. 2020 May 1;74(5):467–72. doi: 10.1136/jech-2019-212208 (PMC7307661; doi:10.1136/jech-2019-212208)
Supplement: Supplementary data [file jech-2019-212208s005.pdf]

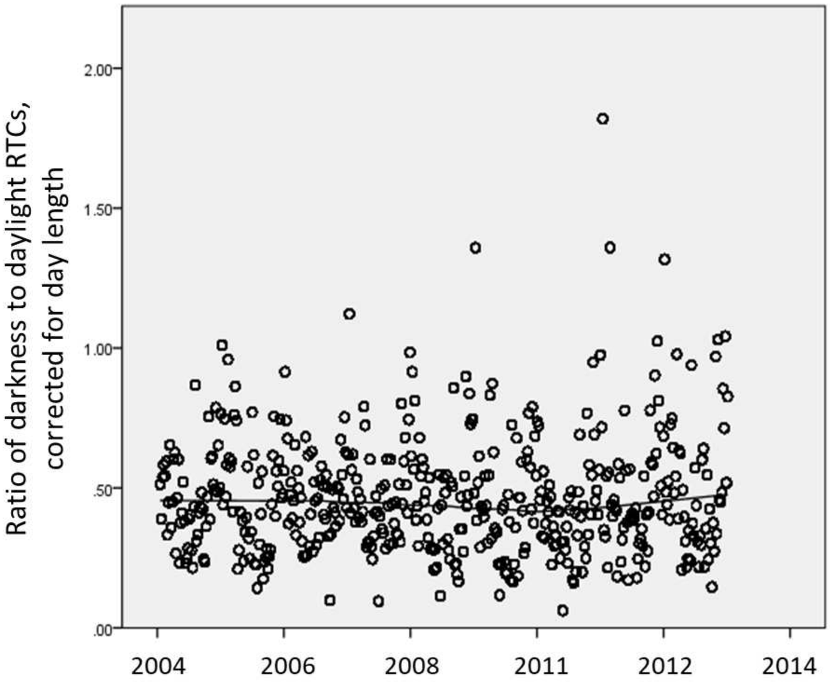

Figure S5. Time series plot of the ratio between time-exposure compensated darkness to daylight RTC rates for the whole City, with an Epanechnikov (40%) smoother put through the ratio data.
